# Role of the Circadian Clock “Death-Loop” in the DNA Damage Response Underpinning Cancer Treatment Resistance

**DOI:** 10.3390/cells11050880

**Published:** 2022-03-03

**Authors:** Ninel Miriam Vainshelbaum, Kristine Salmina, Bogdan I. Gerashchenko, Marija Lazovska, Pawel Zayakin, Mark Steven Cragg, Dace Pjanova, Jekaterina Erenpreisa

**Affiliations:** 1 Cancer Research Division, Latvian Biomedicine Research and Study Centre, LV-1067 Riga, Latvia; ninela.vainselbauma@biomed.lu.lv (N.M.V.); Latvia; salmina.kristine@gmail.com (K.S.); marija.lazovska@biomed.lu.lv (M.L.); pawel@biomed.lu.lv (P.Z.); dace@biomed.lu.lv (D.P.); 2 Faculty of Biology, University of Latvia, LV-1050 Riga, Latvia; 3 R.E. Kavetsky Institute of Experimental Pathology, Oncology and Radiobiology, National Academy of Sciences of Ukraine, 03022 Kyiv, Ukraine; bigerash63@gmail.com; 4 Centre for Cancer Immunology, School of Cancer Sciences, Faculty of Medicine, University of Southampton, Southampton SO16 6YD, UK; m.s.cragg@soton.ac.uk

**Keywords:** cancer resistance, genotoxic treatments, circadian clock (CC), cell cycle, DNA damage response (DDR), reversible polyploidy, reprogramming, senescence, telomeres, Hayflick limit

## Abstract

Here, we review the role of the circadian clock (CC) in the resistance of cancer cells to genotoxic treatments in relation to whole-genome duplication (WGD) and telomere-length regulation. The CC drives the normal cell cycle, tissue differentiation, and reciprocally regulates telomere elongation. However, it is deregulated in embryonic stem cells (ESCs), the early embryo, and cancer. Here, we review the DNA damage response of cancer cells and a similar impact on the cell cycle to that found in ESCs—overcoming G1/S, adapting DNA damage checkpoints, tolerating DNA damage, coupling telomere erosion to accelerated cell senescence, and favouring transition by mitotic slippage into the ploidy cycle (reversible polyploidy). Polyploidy decelerates the CC. We report an intriguing positive correlation between cancer WGD and the deregulation of the CC assessed by bioinformatics on 11 primary cancer datasets (rho = 0.83; *p* < 0.01). As previously shown, the cancer cells undergoing mitotic slippage cast off telomere fragments with TERT, restore the telomeres by ALT-recombination, and return their depolyploidised offspring to telomerase-dependent regulation. By reversing this polyploidy and the CC “death loop”, the mitotic cycle and Hayflick limit count are thus again renewed. Our review and proposed mechanism support a life-cycle concept of cancer and highlight the perspective of cancer treatment by differentiation.

## 1. Introduction

Resistance to anticancer treatments remains a significant problem in medicine and society due to the high morbidity of cancer patients. In 1968, the first clinical report on the association of polyploidy with the progression of malignancy appeared [1]. The chronological landmarks in the field of cancer polyploidy from Virchow and Boveri in the 19th century until now have recently been excellently reviewed [2]. From this perspective article, we build on this framework to evaluate the current knowledge of the role of the circadian clock (CC) in genome deregulation under a lens of its relationship to cancer polyploidy and resistance to anticancer treatments.

It is established that malignant tumours are characterised by various degrees of aneu-polyploidy emerging from whole-genome duplications (WGD) that appear early in cancer evolution, progress with disease aggression, and correlate with resistance to anticancer treatments [3,4,5,6,7]. The first-line anticancer therapies (ionising radiation and genotoxic drugs) kill most tumour cells in the first days after administration. However, these cells can also evoke transient polyploidy that can give rise to clonogenic depolyploidised survivors several weeks or months after treatment cessation, recovering mitotically cycling cells, which disseminate, cause metastases, and can repeatedly polyploidise with disease relapse [8,9,10,11,12]. Observations showed that advanced tumours paradoxically acquire this additive mechanism of clonogenic survival within a certain interval of increasing genotoxic challenge, proportional to induced polyploidy ([12,13,14,15] and unpublished observations). We previously suggested that for both intrinsic and therapy-induced cancer polyploidy, the two reciprocally joined reproduction cycles, the rapid mitotic cycle, and the slow polyploidy cycle can drive cancer cell immortality akin to the transmission of generations in unicellular organisms and termed it the “cancer cell life-cycle” [16,17,18]. A similar process was termed by Jinsong Liu the “giant cell cycle”, highlighting its operation in more malignant tumours [19], while Rajaraman termed this process “neosis” accenting the cycling, budding of young offspring from senescing polyploid cancer cells [10,20]. As proof of concept, in a seminal paper, Zhang Weihua et al. [21] showed that a single polyploid giant tumour cell with senescence landmarks can develop metastatic cancer when transplanted in a mouse. Examples of experimental evidence of this mechanism published in the 21st century from 26 laboratories over the world are gathered in Table 1 and a series of relevant reviews [3,4,5,6,7,8,12,20,22,23,24] and most recently in a Special Issue of Seminars in Cancer Biology [25].

The induction or enhancement of polyploidy by cancer progression or anticancer treatments does not only assign the evolutionary advantage of genome multiplication masking lethal mutations [26,27] and provides an option for effective DNA repair [4,14,28,29]. The accompanying aneuploidy is also trading off its advantages and disadvantages (chromosome mis-segregation) by driving clonal selection in cancer genetic contexts [30,31]. However, it may also contribute via whole-genome triploid bridges [26] between diploid and tetraploid generations. A similar bridge by the doubled maternal genome was identified in a proportion of male para-triploid cancer karyotypes and in vitro on HeLa [32,33]. It is important to stress that polyploidy induced in cancer by genotoxic treatment cannot be reduced to only the genome multiplication and re-arrangements, but it is also accompanied by a crucial change in cell biology—the reprogramming of tumour cells to a state of embryonal stemness [11,13,22,34,35,36,37,38] with germline markers [16,18,36,39,40,41,42]. At this point, the polyploidy facet of cancer biology fuses with that of cancer stem cells, currently considered the main mechanism of cancer cell immortality and treatment resistance [43,44].

Then, there arises an interesting question: how much polyploidy and stemness constitute cancer identity and treatment resistance potential in organisms (independently of somatic mutations)?

The first part of this question is addressed in Section 2 of this review through the evaluation of bioinformatic transcriptome studies of polyploidy in normal mammalian tissues [45,46]. These data revealed in the gene ontologies and gene phylostratigraphy of polyploid mammalian tissues already known mechanisms of carcinogenesis and drug resistance, including stemness, but also unexpectedly the suppression of the circadian clock. The latter finding gave impetus to the current perspective.

The other side of the same question, concerning stemness, was addressed in experiments on irradiation-resistant malignant lymphoma and non-cancerous hepatic stem cell cultures reviewed in Section 3. The mutual features of the DNA damage response (DDR) conferring resistance were found in the adaptation of the DNA damage checkpoints leading to polyploidy. Therefore, in Section 4, we discuss the regulation of the embryonic stem cell (ESC) cell cycle checkpoints and DDR—revealing parallels with cancer polyploidy—in particular, the tolerance to DNA damage. In turn, this highlighted the intrinsic link between the paradoxical coupling of accelerated cellular senescence (ACS) creating and tolerating the DNA double-strand breaks (DSBs) with reprogramming.

ACS was first described as irreversible growth arrest in response to oncogenic, genotoxic, and oxidative stress [47]. Senescent cells possess compromised telomeres signalling persistent DNA damage [48]. In cancer, both opposing phenomena, i.e., senescence and stemness, are interacting through the secretome via paracrine and intracellular mechanisms [49,50]—akin to the process of wound healing [2]. However, the relationship of this paradoxical pairing between ACS and reprogramming with reversible polyploidy as the third component of cancer resistance revealed in a number of studies [51,52,53,54,55,56,57,58], also seen in Table 1, remains poorly understood and often overlooked [25]. Therefore, Section 5 reviews the important actors of ACS and mitotic slippage—including the cGAS-STING pathway, which senses soluble cytoplasmic DNA, in turn, reciprocally activating the Hippo pathway. The latter is involved in the regulation of the correct mitotic segregation of chromosomes, stemness, cell fate change, and reciprocally… again ACS. The missing component of this puzzle, the fate of telomeres eroded by ACS, is analysed in Section 6. There we review and illustrate our own data on the transient alternative telomere lengthening in polyploid cancer giant cells (PGCCs), which ensures recombinative restoration of telomeres and the return of telomerase activity in the budding mitotic progeny of clonogenic survivors. Finally, with the knowledge extracted from previous sections under the lens of cancer polyploidy, we approach the main biological oscillator, the circadian clock (CC), in Section 7. We review the data on CC participation in regulating the normal and ESC cell cycle, DDR, telomere elongation, and the eventual Hayflick limit count by the CC through cell cycles. The following Section 8 is devoted to the deregulation of the CC in mammalian polyploidy and cancer and discusses our current study of the correlative link between the deregulation of CC and polyploidy in primary cancers from the TCGA database. As a result of this analysis, we propose in Section 9 a working hypothesis on the deterioration of circadian rhythm through mitotic slippage in the polyploid phase of the “cancer cell life-cycle”, subsequent telomere restoration by ALT, and reset in resistant de-polyploidised offspring of the telomerase-dependent circadian pacing and Hayflick count of the restored mitotic cycle. We finally outline some other relevant perspectives in the field.

**Table 1:** A summary of experimental evidence for anticancer treatment resistance acquired via reversible polyploidisation of mammalian cancer cells (where the species is not indicated, human material was investigated). PGCC—polyploid giant cancer cells.

Cancer Type	Anticancer Treatments	Experiment Type and The Results	Source
Burkitt’s lymphoma Namalwa and Ramos	Ionising radiation (single dose of 10 Gy)	In vitro. DNA flow cytometry of induced reversible polyploidy; separation of >4C DNA by FACS, clonogenicity of the labelled polyploid fraction; detailed microscopy.	[9,59]
Transformed cell lines, cervical carcinoma, renal adenocarcinoma, neuro-blastoma	Ionising radiation, etoposide	In vitro. Computerised video-time-lapse microscopy recording of polyploidisation followed by bursting or budding of small cells restarting mitosis	[10]
Colon carcinoma DHD-K12-TRb (PROb) (rat)	Cisplatin	In vitro. Prolonged observation revealed delayed emergence of a limited number of extensive colonies which originate from polyploid cells, as demonstrated by cell sorting analysis. These colonies are made of small diploid cells which differ from parental cells by increased resistance to cytotoxic drugs.	[55]
Colorectal carcinoma HT 116	Nocodazole	In vitro. Fluorescence-activated cell FACS-purified cells with an 8n DNA content formed colonies that gave rise to a ~2n generation, which was followed by video-microscopy; the plating efficiency was higher for the TP53^−/−^ subline.	[60]
Lymphoblastoma (WI-L2-NS, TK6), Burkitt’s lymphoma (Namalwa)	Ionising radiation (single dose of 10 Gy)	In vitro. Induction of reversible polyploidy upregulates OCT4, NANOG, and SOX2), which facilitate survival suppressed by retinoic acid. Dependence on mutant TP53 status.	[34]
Fibrosarcoma (mouse)	Doxorubicin	In vitro. Induced and isolated single giant cell allografts cause metastatic cancer.	[21]
NK/Ly lymphoma mouse	Vinblastine	In vivo. An increased number of giant cells were induced by vinblastine treatment and observed microscopically in tumour-bearing mice.	[61]
Colorectal carcinoma HCT116 modified lines	H_2_O_2_	Tetraploid cell line established from parental diploid HCT116 via cell fusion revealed the superiority of tetraploidy over p53 for cell survival when compared by cell viability, cell cycle, and apoptotic response to H_2_O_2_ with parental HCT116 and p53- inactivated sublines.	[62]
Breast carcinoma	Ionising radiation (single dose of 4 and 8 Gy)	Ex vivo. patient samples, ionising radiation reprogrammed differentiated breast cancer cells into induced stem cells. They showed increased mammosphere formation and increased tumorigenicity in xenografts. Reprogramming occurred in a polyploid subpopulation of cells, coinciding with re-expression of the transcription factors Oct4, SOX2, Nanog, and Klf4, and could be partially prevented by Notch inhibition.	[13]
Non-small cell lung cancer in patients, NCI-H1299 cell line	Camptothecin, doxorubicin, cisplatin	Ex vivo: Clinicopathological study in patients with locally advanced non-small-cell lung cancer demonstrate that therapy-induced senescent cells following neoadjuvant therapy are prognostic of an adverse clinical outcome. In vitro: polyploid senescent cells represent transition states through which escape preferentially occurs.	[63]
Breast carcinoma T-47D and ZR-75-1	Genotoxic drugs and mTOR inhibitors	In vitro. Inhibition of mTOR signalling prevents the polyploidy/senescence induced by genotoxic drugs and increases cell chemosensitivity.	[64]
Colorectal carcinoma HCT-116 and Caco-2 cell lines	5-fluorouracil and oxaliplatin	In vitro. CoCl2 induction of hypoxia in colon cancer cells causes the formation of PGCCs, the expansion of a cell subpopulation with CSC characteristics and chemoresistance.	[65]
Virally transformed rat fibroblasts with suppressed apoptosis in E1A + E1B cell lines	Ionising radiation	In vitro. Permanent activation of DDR signalling due to impaired DNA repair results in the induction of cellular senescence in E1A + E1B cells. However, irradiated cells bypass senescence and restore the population by dividing cells, which have a near-normal size and ploidy and do not express senescence markers.	[66]
Ovarian adenocarcinoma, breast carcinoma (HEY, SKOv3, and MDA-MB-231)	Cisplatin	In vitro and in vivo. Separation of induced PGCCs by CoCl2; characterisation of stemness, observation of budding offspring, A single PGCC formed cancer spheroids in vitro and generated tumorigenic xenografts.	[11]
Multiple human tumour types	Etoposide, doxorubicin, ionising radiation	In vitro and in vivo. Cell lines, time-lapse video microscopy observing budding of survivors from giant tumour cells; tumour xenografts.	[22,38]
Ovarian carcinoma (SKVO3, IGROV-1 cell lines)	carboplatin	In vitro. Generation and depolyploidisation of PGCCs by multipolar divisions and budding (time-lapse life cell imaging). Induction of EMT and senescence markers.	[67]
N-RA(61K)-mutant pigment cell culture cell	Doxycycline-inducible activation of oncogenic N-RAS	In vitro. Multinuclear senescent cells are induced, giving rise to mononuclear tumour progeny observed by time-lapse microscopy. The progeny is tumorigenic in xenografts.	[68]
Colorectal carcinoma (HC116)	Doxorubicin	In vitro. The cells which, along with therapy-induced senescence, undergo polyploidisation are prone to regaining the ability to proliferate.	[53]
Ovarian carcinoma (Hey, SKOV3, OVCAR433)	Paclitaxel	In vitro. Generation of genomically altered tumour-initiating cells through a giant cell cycle that contributes to tumour relapse was observed using live-cell fluorescence time-lapse microscopy. PGCCs were shown to self-renew via endoreplication and divide by nuclear budding or fragmentation.	[69]
Breast carcinoma	Doxorubicin + paclitaxel	Ex vivo. Sampling before and after neoadjuvant therapy. Induction of depolyploidising PGCCs positive for OCT4, SOX2, NANOG, and CD44 was mainly observed in near-triploid resistant cases.	[70]
Ovarian carcinoma (Hey, SKOV3, and MDA-HGSC-1 cell lines)	Paclitaxel	In vitro and in vivo. The obtained single PGCCs formed spheroids with the properties of blastomeres, including differentiation into three germ layers and formation of carcinoma, germ cell tumours, as well as benign tissue, in xenografts.	[37]
Prostate carcinoma PC3 line	Docetaxel	In vitro. A micro-fabricated “evolution accelerator” environment for controllable in vitro with a spatially varying drug concentration. The authors observed the rapid emergence of a large number of PGCCs with EMT marks at a very high drug concentration.	[15]
Glioblastoma T98G, A172, R2, T1 cell lines	Ionising radiation; Fotemustine	In vitro. The resistant cell lines displayed the PGCCs and high activity of tumour and microenvironment promoting genes.	[71]
Breast carcinoma and mouse melanoma	5-fluorouracil	In vitro and in vivo. The authors found IL 33 to be a key driver of cancer resistance through polyploidy.	[72]
Breast carcinoma (MDA MB 231 cell line)	Doxorubicin	In vitro. Resistant reversible polyploidisation registered by DNA cytometry; 7-week follow-up; IF, microscopy. Transient ALT in mitotic slippage; Budding of mitotic progeny from PGCCs.	[73]
Ovarian carcinoma (SCOV-3 and A2780 cell lines)	Cisplatin	In vitro. Bioinformatic analysis of induced PGCCs—upregulation of genes mainly related to gene regulatory mechanisms and nuclear processes, including negative chromatid segregation, microtubule polymerization and membrane budding.	[74]

## 2. Transcriptome Analysis of Polyploidy versus Diploidy in Normal Mammalian Tissues Reveals a *c-Myc*-Targeted Shift to Stemness and Other Known Mechanisms of Cancer Origin and Resistance

When the polyploid transcriptomes of normal mammalian tissues (heart, liver) are compared with diploid cells, the upregulation of *c-Myc*—an essential component of Yamanaka reprogramming [75]—is evident [45]. To evaluate its impact, a bioinformatic comparative study focused on multiple primary targets of *c-Myc,* alongside gene phylostratigraphic analysis of transcriptomes, was performed [46]. Surprisingly, they revealed in the gene ontologies of differentially the expressed genes (comparing polyploid and diploid) the already known mechanisms of cancer and drug resistance, such as the Warburg effect, the epithelial-mesenchymal transition (EMT), alongside atavistic features of unicellularity originating the ABC drug efflux, suppression of apoptosis, differentiation and cellular communication, immune evasion, enrichment of bivalent-chromatin genes, and suppression of the CC.

## 3. Resistance to Ionising Irradiation in Malignant Tumours and Tissue Stem Cells Is Associated with Induced ESC Stemness Concurrent with Senescence, Weak DNA Damage Checkpoints, and Polyploidy

The normal mitotic cell cycle consists of the G1, S, G2 and M phases. Progress through these is driven by corresponding cyclin-kinases. If DNA damage has occurred, cells can activate the G_1_, intra-S, and G_2_/M checkpoints and arrest the cell cycle to repair the damage. There are the two major DNA damage signalling pathways—regulated by ATM/CHK2 and ATR/CHK1. The ATM/CHK2 pathway is primarily activated by double-strand breaks (DSBs), while the ATR/CHK1 pathway is triggered in response to replication fork collapse. Following DNA double-strand breaks (DSB), the ATM protein is activated by autophosphorylation, which then activates CHK2. The p53 tumour suppressor, a major effector of the DDR pathway, is expressed at low levels and in an inactive form during normal conditions. Both ATM and CHK2 phosphorylate p53, causing its stabilisation and activation. Activated p53 arrests the cell cycle by inducing cell cycle inhibitors such as p21/CIP1. The DDR acting at the checkpoints normally allows the cell to repair its damaged DNA or alternatively undergo apoptosis [76].

**Figure 1:**
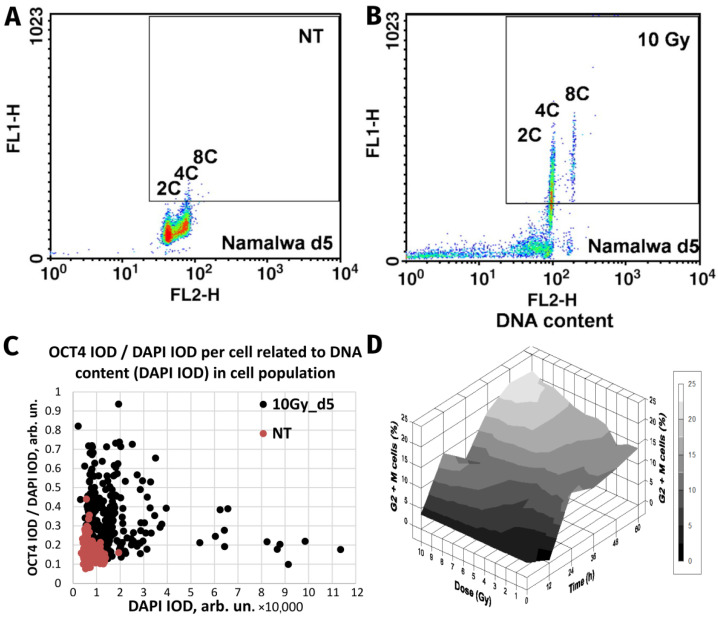
The similarity of responses to acute Irradiation (10 Gy) of the malignant human Burkitt’s lymphoma cell line Namalwa and benign rat liver progenitor stem line WB-F344. Radiation-induced Oct4 upregulation in Namalwa cells as revealed by flow cytometry: panel (**A**) unirradiated cells (control); panel (**B**) irradiated cells on day 5 post-irradiation. According to the extent of the FL1-signal (immunofluorescence from Oct4), Oct4 is predominantly expressed in polyploid 4C and 8C cells whose DNA content was determined by propidium iodide staining for DNA (FL2-signal) (reprinted with permission from [34]. Copyright ID 1188250-1, 2022, Elsevier Science &Technology Journals). Panel (**C**) radiation-induced Oct4 upregulation in WB-F344 cells as revealed by two-parametric image analysis of integral optical densities (IOD): represented as Oct4 (IOD)/DAPI (IOD) versus DAPI (IOD). Panel (**D**) radiation-induced G2/M delay in WB-F344 cells which is dose- and time-dependent (image from [78]).

Whereas normal healthy somatic cells have the fate indicated above, the response of malignant cancer cells can differ, leading to treatment resistance. Current data suggest that resistance can be induced in malignant tumour cells by reprogramming to an ESC-like state accompanied by WGD [13,34,77]. As illustrated in Figure 1A,B, the master regulator of embryonic stemness OCT4 can be induced in the mtTP53 Burkitt’s lymphoma cell line, Namalwa, alongside polyploidisation after 10-Gy irradiation [34]. In our study, the upregulated OCT4 alongside Nanog and SOX2 were shown to create a coordinated nuclear network, while all-trans-retinoic acid (an OCT4 antagonist and differentiation inducer) disrupted the nuclear localisation of Nanog, and subsequently cell survival. Similarly, the embryonal-type stemness could be partially suppressed by Notch inhibition [13,77].

Strikingly similar post-irradiation effects were found in the rat liver cell line WB-F344, which is a hepatic tissue-specific stem cell line capable of differentiating into hepatocytes and cholangiocytes [29]. This wtTP53 cell line, benign and incapable of inducing tumours in vivo, was shown to be radioresistant [78]. In common with genotoxically resistant cancers, the prominent feature of WB-F344 cells is a radiation dose-dependent enhancement of polyploidisation and micronucleation [29,78]. In this study, along with polyploidisation, there was also upregulation of the stemness transcription factors Oct4 and Nanog following 10-Gy irradiation [29], particularly enhanced in the polyploid fraction (Figure 1C). Thus, while one cell type is malignant (Namalwa) and the other benign (WB-F344), both radioresistant cell lines are similarly capable of the DNA damage-induced reprogramming—evoking the induction of ESC-type stemness alongside polyploidy. Finally, there was a radiation dose-dependent delay at the G2/M checkpoint (Figure 1D) that preceded and was proportional to the extent of polyploidisation within a considerable interval of increasing resistance. The same was found for malignant TP53-mutant lymphomas [14,59], as well in prostate cancer and colorectal cancer treated with genotoxic drugs [15,65], and in unpublished research. This response, characteristic for both benign and malignant resistant cell lines, reacting to irradiation with polyploidy-associated reprogramming to an ESC-state, is indicative of: (1) the weakness of the G1 checkpoint resulting in cell accumulation in G2M and, concurrently, (2) the insufficiency of the G2M damage checkpoint, the main DSB sensor and actor, showing the tolerance to DNA DSBs and allowing transition to polyploidy.

In addition, in both models, there is a dynamic toggle between two stemness and senescence regulators, NANOG and p16INK4a [29,34].

Concluding this section, the in vitro studies on irradiation-resistant malignant tumours and tissue stem cells revealed the following consistent features: induction of embryonic-type stemness (reprogramming) concurrent with senescence, attenuation of the DDR and transient polyploidisation. To better dissect this common mechanism, the regulation of the cell cycle checkpoints in ESCs will be reviewed in the next section.

## 4. Embryonic Stem Cells (ESCs) Have Defective Cell Cycle Checkpoints That Favour DNA Damage Tolerance and a Shift to Polyploidy

There is a body of evidence indicating that ESCs have a short G1-phase and weak or absent G1/S checkpoint or a long S-phase and weak intra-S and G2/M checkpoints [79,80,81]. In response to stress, ESCs have a tendency to undergo mitotic slippage from the spindle checkpoint, shifting to G1-tetraploidy at a specific stage with non-degradable cyclin B1, which protects ESCs from mitotic catastrophe [82]. In irradiated tumour cells, this stage may be preceded by delayed endo-prometaphase [83]. Under stress, ESCs epigenetically switch off the genome-guardian function of p53 [79,80]. The same is known for even TP53 wild-type tumours [84]. Presumably, the inherent risk of genome instability that this brings is offset by and required for their strategy for survival reliant upon explorative adaptation, which demands the freedom of choice [16,85]. The induction of stemness in the damaged tumour cells in many ways is akin to the induced reprogramming by Yamanaka factors in normal cells [75], which also simultaneously causes DNA damage-tolerating senescence [48] that paradoxically is indispensable for its induction [50].

**Figure 2:**
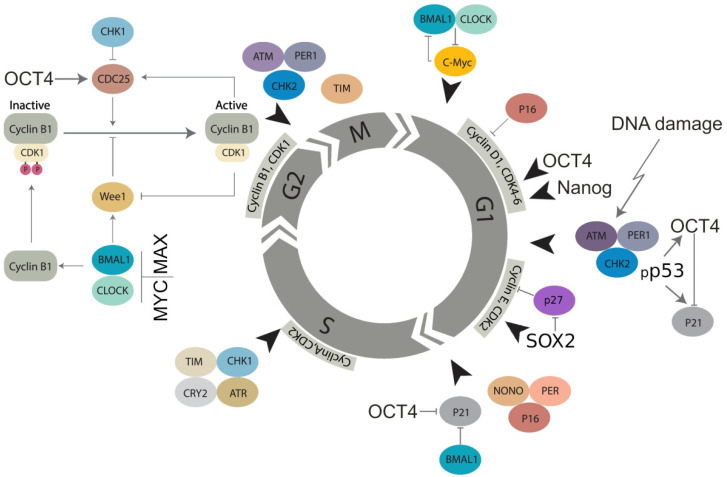
Molecular linkage between the regulators of the cell cycle in embryonal (cancer) stem cells with the checkpoints adapted by basic stemness transcription factors in their relationship with CDK inhibitors (not all of them are shown) and the circadian clock (adapted from [94] under Creative Commons Licence). The details of the action of the circadian clock regulators in DNA damage checkpoints and WGD are reviewed in Section 7 and Section 8.

Transcription factors of the basic embryonal stemness network also possess the properties of cyclin-kinases or can otherwise overcome the senescence-driving and cell cycle-arresting cyclin-kinase inhibitors of the corresponding checkpoints. In particular, OCT4 induces the adaptation of the G1/S checkpoint by activating Cdk2 in the Cyclin E/Cdk2 complex [86,87] and enhancing the transcription of cyclin-kinases CDK4 and CDC25A [79,88]. OCT4 also toggles p21CIP1 [89] in a p53-dependent (DDR-induced) manner [56,57,90]. Nanog activates Cdk6 by directly binding by its C-domain [79], thus competing in the G1/S checkpoint with p16INK4a, which inhibits cyclin D. In the DDR, p16 is also activated by exaggerated expression of p21 and can cause terminal senescence [91]. Concurrently, together with IL-6 secreted by senescent cells, p16 is paradoxically indispensable for reprogramming [49]. In turn, SOX2 directly interacts with p27(KIP1) in reprogramming to stimulate adaptation of the Cyclin E/Cdk2-dependent G1/S checkpoint [92] and also restricts the G2M checkpoint [93]. The most important activation of CDKs and opposing interactions between the embryonal stemness factors (OCT4, Nanog, and SOX2) with corresponding senescence regulators (p21, p16, p27) are shown in Figure 2.

This scheme will be used again in Section 7 and Section 8 to describe the role of the CC in the cell cycle, WGD, and cancer. The current analysis indicates that ESCs tend to adapt to the checkpoints of the normal cell cycle, especially as part of their DDR.

“He who dares wins” (*qui audet vincit*). Mitotic slippage (MS) represents a transition compartment between the mitotic cell cycle and polyploidy in tumours undergoing DDR-mediated reprogramming. Three additional issues about MS need to be understood: (1) How the centrosomal cycle is affected? (2) What happens to the telomeres? (3) What is occurring with the biological time upturning from cell senescence for the birth of a new mitotic offspring?

## 5. The Hyperactivated Hippo-YAP Pathway Relieves Control of Karyo-Cytokinesis, Reciprocally Favours MS, ACS, cGAS-STING Signalling and Polyploidy, and Enables Cell Fate Change

The Hippo pathway is an important regulator of genome stability, stem cell biology, and cell fate change [95]. It was also shown to be involved when deregulated in the origin of cancer polyploidy through cytokinesis failure [96,97,98,99,100]. Currently, it appears that the participation of Hippo deregulation in the events around MS is multifaceted, and here, we attempt to consolidate them. Normally, the main effector of the Hippo pathway, YAP1, is retained in its phosphorylated form in the cytoplasm. The nuclear import of the de-phosphorylated YAP1 initiated by LATS1/2 enables its binding in a (YAP1 + TEAD) complex to DNA, which facilitates hyper-transcription and replication stress due to multiple targets of hyperactivated YAP1 [101]. Interleukin-6, a pivotal senescence inducer indispensable for reprogramming [49], is one of these targets [102]. The feedback loop of cellular senescence in Hippo-YAP signalling has also been reported [103]. The ACSs were shown to release heterochromatin particles into the cytoplasm inducing autophagic lysosome activity [104] and production of cytoplasmic DNA. This activates the cytosolic DNA-sensing cGAS-STING pathway, producing diverse interferons and inflammatory cytokines [105,106]. The ACS-associated degradation of nuclear lamin B favours mitotic slippage and micronucleation of such cells, resetting interphase in a tetraploid state [107]. GAS-STING signalling, in turn, causes reciprocal deregulation of the Hippo-YAP1 pathway by inducing its upstream LATS1/2 kinase [108].

On the other side, the Hippo-related Aurora-A-Lats1/2-Aurora-B axis is pivotal for the centrosome cycle and accurate coordination between chromosome segregation, karyokinesis, and cytokinesis in anaphase and midbody abscission in telophase. Deregulation of this axis subsequently leads to aberrant metaphases, anaphase bridges, bi-nuclearity, multinuclearity, and fusion of daughter nuclei [96,97,98,99,100]. In addition, the stress-activated LATS1/2 causes the dysfunction of the pivotal guardian of genome stability and diploidy p53 [109] and thus can compromise its ploidy control [110].

The coordinated functions of the Hippo pathway ensure genome stability, whereas stress-induced dysfunction likely creates a vicious cycle through reciprocal activities and feedback loops starting with replication stress and around MS, which favour the transition of cancer cells to polyploidy with all its attributes—stemness, ACS and aneuploidy. Moreover, the stress-response is preceded by fast (0.5–2 h) oscillations of YAP1 nucleo-cytoplasmic localisation [111]. Interestingly, in response to DNA breaks induced by irradiation of MCF7 cells, p53 also oscillates in the p53-MDM2 loop with a similar periodicity [112] and drives the OCT4-p21CIP1 stemness-senescence toggle in embryonal carcinoma [56,57]. Cells that experience p53 oscillations recover from DNA damage, whereas cells exposed to sustained p53 signalling have poor survival [113]. It is tempting to suggest that both pulsing activities of two main tumour suppressors and genome guardians, when induced by lethal genotoxic challenge, coordinate their oscillations. In thermodynamic terms, such oscillations between the opposite genome and cell state favour explorative adaptation to the immediate alternative microenvironments to increase the chance of survival [16,114]. However, it is not immediately clear how this vicious circle solves the telomere problem of ACS in cancer resistance.

## 6. Under-Replication, Erosion, and Recovery of ACS-Compromised Telomeres in Mitotic Slippage and Transient Polyploidy through Transient Alternative Telomere Lengthening

Cancer cell lines undergoing mitotic slippage accompanied by the cytoplasmic release of chromatin after genotoxic challenge also exhibit the under-replication of DNA in the late S-phase [57,73]. Under-replication of heterochromatin has been widely described in plants and insects, and Walter Nagl [115] indicated that it was always and only associated with the endocycle. Recent studies on Drosophila polyploid cells associate telomere under-replication with inhibition of replication fork progression and control of DNA copy number [116,117].

**Figure 3:**
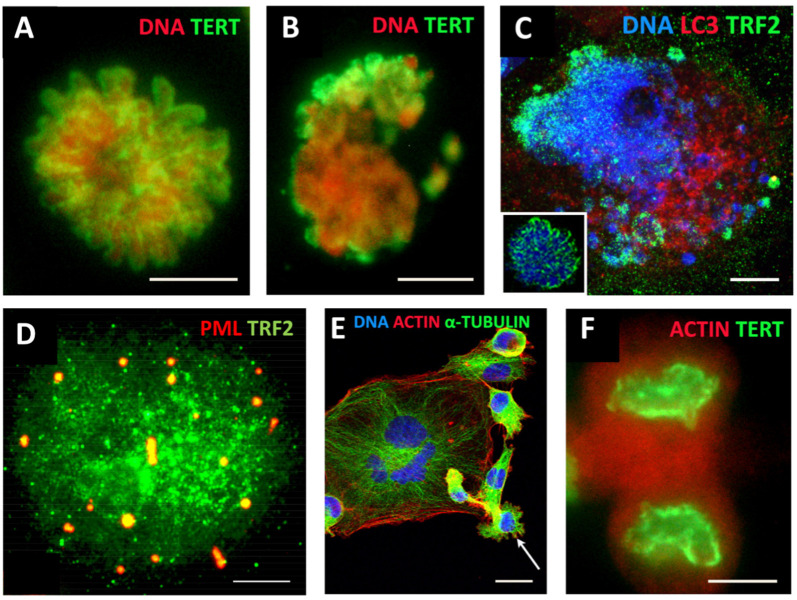
Mitotic slippage of the MDA-MB-231 breast cancer cell line. (**A**) TERT-positive metaphase in control cells (DNA counterstained by propidium iodide); (**B**) mitotic slippage with low TERT nuclear and enriched cytoplasmic DNA staining on Day 5 after DOX treatment; (**C**) preferential release of the telomere shelterin-TRF2-associated chromatin into the cytoplasm on Day 7 after DOX treatment (insert: normal metaphase); (**D**) polyploid cell marked by specific TRF2-positive PML bodies, suggesting the restoration of the telomeres by alternative telomere lengthening (ALT); (**E**) A giant multinuclear cell is budding subcells (arrow); (**F**) TERT-positive escape telophase cell on Day 22 after DOX treatment; Bars: (**A**–**D**,**F**) = 10 µm; (**E**) = 25 µm. Subfigures A–C,E,F are republished from [73] under Creative Commons Licence.

ACS was defined by Campisi [118] as cell stress that is characterised by compromised shortened telomeres, which may be induced by oncogenic stress or DNA damage. As such, it appears that telomere erosion stemming from heterochromatin under-replication may, in fact, result from the replication stress observed in cancer development and treatment [119], occurring in the S-phase preceding polyploidisation by MS in the same or rather (as observed) next cell cycle (involving the relaxation of the “Hippo-genome-stability barrier” by YAP1-hyperactivation as already discussed). Tam et al. [120] likely were the first to define ACS as a reversible process that is determined by the balance of biological molecules which directly or indirectly control telomere length and telomerase activity by altering gene expression and/or modulating the epigenetic state of the chromatin. Our studies on the MDA-MB-231 breast cancer cell line treated with the Topoisomerase II inhibitor doxorubicin [73] revealed telomere ends enriched in DSBs were discarded during MS together with the telomere capping protein TRF2 and the telomerase catalytic subunit TERT (Figure 3A–C). In the inter-and post-MS polyploid cells, restoration of the telomeres by alternative telomere lengthening (ALT) marked by specific TRF2-positive PML bodies was found (Figure 3D). It was followed by the recovery of TERT activity in the budding offspring returning to the mitotic cell cycle (Figure 3E,F). Importantly, in this interim process of telomere restoration through ALT-driven homologous recombination, the telomere ends of the chromosomes were found closed [73]. Telomere shortening in diploid somatic cells is associated with the linear chromosome end replication problem, cutting telomeres in each cell cycle by ~50 bp [121]. This process is the molecular basis underpinning the Hayflick limit [122], permitting somatic cells to replicate only a limited number of times, proportional to the species’ lifespan. Thus, with the “trick” of under-replication signalling ACS and transient ALT, the chromosome end problem and the Hayflick (somatic mortality) limit may be circumvented by polyploid tumour cells.

A positive regulator of telomere length, Sirtuin 1—a NAD-dependent histone deacetylase (HDAC)—binds directly to telomere repeats and attenuates telomere shortening associated with mouse ageing; this effect is dependent on telomerase activity [123]. At the same time, SIRT1 is very tightly associated with the regulation of the main cellular pacemaker—the CC. To analyse this aspect, we must first briefly describe the inner workings of this remarkable clock in the normal and ESC cell cycle (the latter is induced in tumours by DDR as described above in Section 4).

## 7. The Circadian Clock (CC) Paces the Mitotic Cell Cycle, DDR Checkpoints, and Reciprocally, the TERT-Dependent Hayflick Limit Count. It Is Absent in ESC, Early Embryo, and Germ Cells and Likely Becomes Dis-Engaged and Then Restored (By Reversible Polyploidy) in Cancer Cells

The bi-phasic CC is an autoregulatory transcriptional feedback loop-based oscillator involved in pacing the processes of living organisms with 24 h periodicity [124]. The CC also regulates the cell cycle and couples various metabolic oscillations with shorter ultradian periodicity [125].

The core structure of the CC’s molecular oscillator contains a transcriptional activator, made up of *BMAL1* and *CLOCK*, and a transcription repressor consisting of *PER* (Period) and *CRY* (Cryptochrome) genes. The heterodimeric complex of BMAL1 and CLOCK, which are basic helix-loop-helix transcription factors, binds the promoters and activates the expression of PER1, PER2, PER3, CRY1, and CRY2, which, in turn, heterodimerize into PER/CRY complexes, translocate into the nucleus, and repress BMAL1/CLOCK [126]. The concentration of PER and CRY proteins is regulated by E3 ubiquitin ligases, resulting in their eventual depletion and BMAL/CLOCK1 reactivation [127]. A second, adjacent feedback loop involves nuclear receptors that bind DNA in a periodic manner—the activating RORs and repressive REV-ERBs [128]. These nuclear receptors regulate the expression of BMAL1 and NFIL3 and are themselves rhythmically regulated by the action of NFIL3, CLOCK, BMAL1, and DBP. In this way, the expression patterns of the clock components induce oscillatory behaviours in their downstream interactants [94,124]. It was also shown that alternative splicing, as well as piRNA-mediated regulation of the transposons, could represent another level of clock control [129]. The CC, in general, is susceptible to stress—the circadian cortisol-mediated entrainment of ultradian transcription pulses that provide the normal feedback regulation of cellular function is then lost [130].

Several genes of the CC deliver the strictly synchronised oscillation frequencies of the cell cycle [94,131] and participate in the regulation of the DNA damage checkpoints [125,132], as presented in Figure 2 [94]. The CC becomes dysfunctional in reprogramming induced by Yamanaka transcription factors [133]. Interestingly, circadian oscillation is also not detectable in ESCs until differentiation starts [134]. This may be related to the overexpressed stemness transcription factors speeding the cell cycle and forcing adaptation of its checkpoints, as discussed in Section 4 and illustrated in Figure 2. In addition, the direct competition of the main reprogramming transcription factor, MYC/MAX, with the CLOCK/BMAL1 dimer [125] in the G1/S and G2M checkpoints [135], which can be overcome through upregulated MYC [136] (as designated on Figure 2), should be highlighted.

The loss of circadian rhythms impairs Hippo signalling, destabilises p53 [137], and potentiates tumour initiation [138]. On the contrary, in vitro differentiation of ESCs induces cell-autonomous robust circadian oscillation [139]. It is important to note that besides ESC, the CC is also not functional in normal primordial germ cells (PGCs) and both male and female gonocytes [140,141,142]; the germline-specific protein PIWIL2 suppresses circadian rhythms [143] by inactivating the *BMAL1* and *CLOCK* genes.

Noteworthy, in mammalian sperm, the telomere ends are joined, forming looped chromosomes [144], such as those observed in mitotic slippage of cancer cells [73] and also in bi-parental bi-chromatid genome segregation found by us alongside conventional mitoses in ovarian embryonal carcinoma [145]. Interestingly, early mammalian embryos also display segregation of biparental genomes in the first short cleavage cycles [146] and also lack circadian regulation, which initiates in late embryos, tightly coupled to cellular differentiation (in particular, somitogenesis) [147].

The above-mentioned telomere-specific nicotinamide adenine dinucleotide (NADþ)-dependent HDAC SIRT 1, maintaining telomeres through telomerase activity, was found to interact with CLOCK and to be recruited to circadian promoters in a cyclic manner [148]. In particular, Wang et al. [149] showed that *Sirt1*-deficient mice exhibited profound premature ageing and enhanced acetylation of histone H4 in the promoter of *Per2*—the latter leads to its overexpression. In turn, Per2 suppresses *Sirt1* transcription through binding to the *Sirt1* promoter at the Clock/Bmal1 site. This negative reciprocal relationship between SIRT1 elongating telomeres and CC pace observed also in human hepatocytes [150] may perform the Hayflick limit count by CC.

We can subsequently rationalise that telomere shortening in ACS slows the circadian time-count, and further interruption of telomerase maintenance by TERT in MS substituted by recombination-based ALT with closed telomere ends should interrupt CC (arresting the biological time pace) while returning to the TERT mechanism in depolyploidised offspring restoring the mitotic cycle [73] should resume the CC oscillator and hence the Hayflick limit count. This manipulation of biological time in MS is reminiscent of a “death loop” in aviation.

## 8. The Circadian Clock Is Deregulated in Mammalian Polyploidy and Cancer

### 8.1. The Reciprocal Regulation of Polyploidy and CC Activity in Non-Malignant Tissues

The competitive antagonism of the overexpressed stemness/reprogramming master factor dimer MYC/MAX with CLOCK/BMAL1, which is the core component of the CC’s activation arm and a regulator of the G2M DNA damage checkpoint, is likely to play a key role in impairing the CC in stem cells (including stressed cancer cells that have undergone reprogramming), where stemness features were shown to be tightly coupled to deregulation of the cell that leads to polyploidy. The Timeless (TIM) gene was shown to be involved in the S-phase checkpoint [76]. The circadian clock proteins PER1, PER2, and PER3 are involved in the ploidy regulation of non-cancerous liver cells, and their inactivation results in rampant polyploidisation (both in terms of polyploidisation frequency and increased ploidy counts in the polyploid hepatocytes) [151]. It is also important to mention that of the 16 core genes of the circadian clock (*CLOCK, ARNTL (BMAL1), ARNTL2, NPAS2, NR1D1, NR1D2, CRY1, CRY2, DBP, TEF, RORA, RORB, RORC, PER1, PER2*, and *PER3*) [152,153] 50% are bivalent genes [154] allowing rapid cell fate change. Interestingly, polyploidy (the endocycle) in plants was shown to decelerate the circadian rhythm [155]. In contrast, evidence from mouse and human transcriptome analyses suggests that the deregulation of the CC promotes polyploidisation and vice versa [46,151]. In turn, polyploidy in normal tissues, such as the mammalian heart and liver, is associated with upregulated *c-Myc* and the stemness and cancer-linked EMT targets [45]. The role of the CC in cell cycle integrity and DDR signalling is further showcased by its involvement in DNA repair after ionising irradiation damage (by inducing DDR-signalling genes) (Figure 2) [94,156,157].

The CC was reported as notably dysregulated in cancer [152,153,158], and perturbation of the CC is in itself carcinogenic [159,160]. Meta-analysis of 7476 cancer cases from 36 sources [161] revealed that low expression of PER1 and PER2 correlates with poor differentiation, worse TNM stage, metastases, and reduced patient survival.

Overall, the currently available information on the connection between the CC, stemness, and the cell cycle, as well CC deregulation in cancer, leads us to suggest that circadian dysregulation in human cancer may be largely associated with its polyploidy component as it is in normal mammalian heart and liver. In the next section, we describe an attempt to investigate this hypothesis through bioinformatic analysis of primary cancers.

### 8.2. Circadian Deregulation Correlates with Polyploidisation (Whole-Genome Doubling) in Malignant Tumour Patient Samples

In order to investigate the possible connection between polyploidy and CC deregulation in cancer, it was first necessary to calculate the measure of circadian deregulation. To that end, we used the Cancer Genome Atlas (TCGA), a large-scale collection of omics and clinical data on over 30 types of malignancies from over 11,000 patients [162]. TPM-normalised Rsubread-processed TCGA gene expression data were obtained from the GSE62944 GEO dataset [163]. In order to ensure statistical power, only TCGA transcriptomics datasets counterpart by at least 35 available normal samples were selected, resulting in a final cohort of 11 cancer types and 6667 samples (613 normal and 6054 tumours).

Circadian deregulation in TCGA cancer samples was determined using the CCD method and deltaccd R package developed by Shilts et al. [153], which compares core CC gene co-expression (Spearman rank-based correlation) between samples used in the study and a pan-tissue reference matrix calculated from eight normal mouse datasets with available time data. The Euclidean distance between CC gene correlation vectors of the samples and the mouse reference is referred to as the Clock Correlation Distance (CCD). The difference between normal vs. reference CCD, and the tumour vs. reference CCD, known as the ΔCCD, serves as a coefficient of circadian dysregulation, with the “difference of differences” approach effectively negating the nuance of mouse–human comparison and accepting the common regulation of CC in mammals [164].

Tumour ploidy calculated from somatic DNA alteration data using the ABSOLUTE algorithm [165] was obtained from [166], and the relationship between the values of scaled ΔCCD for each of the 11 tumour types and the respective proportion of samples with at least one WGD was investigated using Spearman correlation analysis.

**Figure 4:**
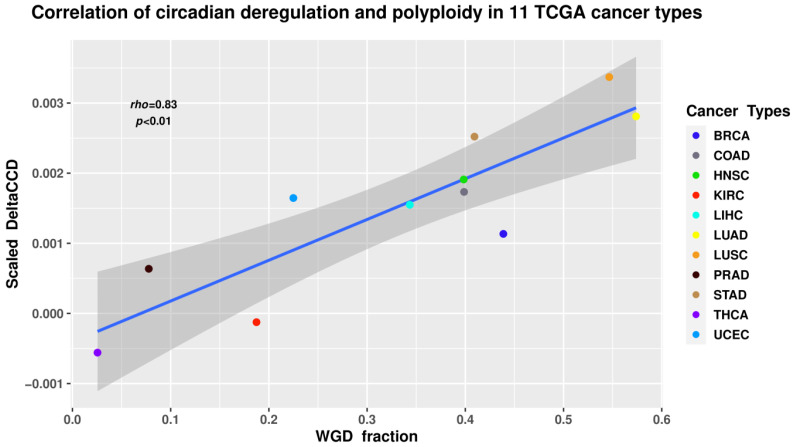
The ΔCCD coefficient of circadian deregulation positively correlates with the proportion of WGD in the samples of 11 tumour types from The Cancer Genome Atlas (TCGA) database. BRCA—breast carcinoma; COAD—colon adenocarcinoma; HNSC—head and neck squamous cell carcinoma; KIRC—kidney renal cell carcinoma; LIHC—liver hepatocellular carcinoma; LUAD—lung adenocarcinoma; LUSC—lung squamous cell carcinoma; PRAD—prostate adenocarcinoma; STAD—gastric adenocarcinoma; THCA—thyroid carcinoma; UCEC—uterine corpus endometrial carcinoma.

The results revealed a statistically significant positive correlation (Spearman’s rho = 0.83; *p* < 0.01) between WGD and CC deregulation (Figure 4). While correlation does not necessarily equal causation, such a result seems logically sound when taking into account the known associations between polyploidy and the CC in normal tissues, deregulation of CC in cancers, as well as the impact of polyploidy on cancer evolution.

## 9. Conclusions, Hypothesis, Perspectives

**Figure 5:**
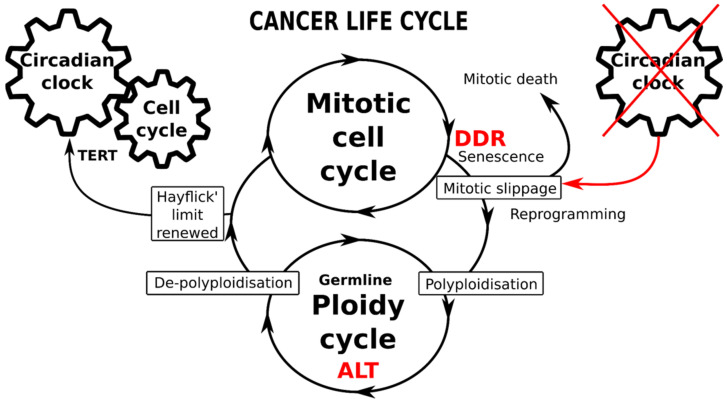
Schematic of the immortal cancer life-cycle composed of two reciprocally joined mitotic and ploidy cycles. The mitotic cell cycle is driven by the circadian clock (CC), particularly operating the telomerase-dependent telomere maintenance pathway (TERT). The transition from mitotic to ploidy cycle occurs after DNA checkpoints are adapted during the DNA damage response (DDR), through mitotic slippage coupling accelerated cellular senescence (with compromised telomeres) and reprogramming to whole-genome duplications. Transition into the ploidy cycle, concurrent with a germline expression signature, is associated with interruption of the circadian clock and restoration of eroded telomeres by alternative telomere lengthening (ALT). Return of depolyploidised offspring to the mitotic cycle restores the TERT-pathway and the CC-driven count of the Hayflick limit.

Currently, data regarding the role and importance of circadian rhythms deregulation in cancer are accumulating. In this perspective article, we have attempted to untangle the involvement of this basic biological oscillator in the processes associated with one of the hallmarks of cancer aggression and resistance—aneu-polyploidy and its association with ACS and reprogramming. On the basis of our analysis, we propose a working hypothesis presented in Figure 5.

The adaptive response to genotoxic, oncogenic or oxidative stress induces ACS with telomere attrition coupled and toggled with stemness (reprogramming). While the former produces persistent DDR signalling, the latter concurrently attenuates the DDR checkpoints and supports DNA damage tolerance; such coupling is thus potentially redirecting stressed cancer cells from the mitotic cycle through mitotic slippage into polyploidy. The cell cycle drivers and DDR checkpoints are robustly regulated by CC genes; however, they become compromised by mitotic slippage into polyploidy (or even earlier, in the preceding replication stress). The data show that to get into transient polyploidy after receiving genotoxic stress, a cancer cell should perform a “death loop”—first, by falling out of the conventional mitotic cell cycle, driven by the CC, into a polyploidy cycle with a decelerated or dysfunctional CC and then undertaking a return to the mitotic cycle, re-engaging the CC to count the replication life-span again. This critical transition from the mitotic cycle into transient polyploidy appears focused on mitotic slippage interrupting the circadian regulation. The return to the normal biological time pace, which is associated with counting the Hayflick limit, needs the eroded telomeres to be restored and linked again to a telomerase-dependent mechanism (TERT). This telomere restitution mechanism may be performed by ALT coupled to a kind of meiotic homology search and recombination [73]. In some way, the “fall” into transient polyploidy resets the cell to a “timeless state”, the likes of which are normally displayed only by germ cells and early embryos which lack CC oscillation. It is noteworthy that cancers [40,167] and PGCCs, in particular, abundantly express the germline genes and proteins [39,41,42,83,168]. Furthermore, the connection between the mitotic slippage-induced cGAS-STING pathway and one of its targets—the transmembrane protein family Fragilis, is involved in the commitment of primordial germ cells and oogonia [169,170] may be also involved in this soma-to-germ transition. In summary, these current data on the CC suggest possible participation in cancer treatment resistance and provide an additional argument in support of the oldest embryonic concept of cancer with its parthenogenetic and parasexual variants [17,32,33,36,37,171,172,173]. Within this logic, the various requirements of the above-discussed mechanisms of resistance to anticancer treatments may be provisionally met.

How can this knowledge be further used? Which drawbacks and perspectives for cancer research and treatment are illuminated?

In Section 5, we evaluated the pathways at play during mitotic slippage, particularly those surrounding the mitotic-to-polyploidy transition with its still poorly understood cross-talk between ACS and the cGAS-STING and Hippo pathways. We highlighted the induced oscillation of two pivotal genome stability guardians—the Hippo and p53 pathways, both components of the genotoxic stress-response and occur with a similar periodicity. In fact, these represent oscillations between senescence inducing DSBs and stemness, relaxing the DDR response and interfering with the CC regulation of the cell cycle. It may be speculated that these combined oscillations of the two pathways create fluctuation in a coherent mode to push cells from the conventional mitotic path into the new cell state of the polyploidy cycle uncoupled from the CC. Here, the laws of unstable thermodynamics of open systems [174] are acting. From the methodical point of view, such regulation by oscillation between opposing states needs a further appreciation of the circular causality implicit in any feedback process within certain parameters [114,175] and demands the design of dynamic studies on individual cells. Such a stress response incorporating an explorative oscillatory adaptation with a critical transition into the PGCC acquiring the germline or embryo-like potential, and a similar “timeless” CC state dangerously increases the chance of cancer relapse after aggressive genotoxic modalities [12,16,114,176].

However, the good news is that the initiation of cell differentiation in ESCs induces an autonomous circadian rhythm [139] that, in turn, drives a normal cell cycle from one checkpoint to another like a good operator drives a train from one station to the next. It currently appears, therefore, that instead of killing cancer cells with genotoxic treatments, the strategy of cancer normalisation by differentiation is more prospective. The embryonic features of PGCCs provide a fundamental basis for the epigenetic reversion of malignancy [36,37,177]. In such modalities, epigenetics can overcome genetics [178,179,180]. The various tumour-differentiation strategies, including environmental 3D structurisation, have been suggested and undergo testing [179,181,182]. The chronotherapy concept [183,184] may be considered in combination with differentiation-inducing modalities. In addition, as shown by our bioinformatic analysis, the measure of CC deregulation correlated with polyploidy obtained from transcriptome or other methods could have potential in new cancer diagnostic and prognostic settings.

## Data Availability

The reprocessed TCGA cancer and the normal transcriptomic dataset are available in the GEO repository under the GSE62944 accession number. TCGA tumour ploidy data calculated by ABSOLUTE from [166] are available in the Supplementary Materials of said study at https://gdc.cancer.gov/about-data/publications/pancan-aneuploidy, accessed on 5 November 2021.
